# Comprehensive prediction of drug-protein interactions and side effects for the human proteome

**DOI:** 10.1038/srep11090

**Published:** 2015-06-09

**Authors:** Hongyi Zhou, Mu Gao, Jeffrey Skolnick

**Affiliations:** 1 Center for the Study of Systems Biology, School of Biology, Georgia Institute of Technology, 250 14th Street, N.W., Atlanta, GA 30318.

## Abstract

Identifying unexpected drug-protein interactions is crucial for drug repurposing. We develop a comprehensive proteome scale approach that predicts human protein targets and side effects of drugs. For drug-protein interaction prediction, FINDSITE^comb^, whose average precision is ~30% and recall ~27%, is employed. For side effect prediction, a new method is developed with a precision of ~57% and a recall of ~24%. Our predictions show that drugs are quite promiscuous, with the average (median) number of human targets per drug of 329 (38), while a given protein interacts with 57 drugs. The result implies that drug side effects are inevitable and existing drugs may be useful for repurposing, with only ~1,000 human proteins likely causing serious side effects. A *killing index* derived from serious side effects has a strong correlation with FDA approved drugs being withdrawn. Therefore, it provides a pre-filter for new drug development. The methodology is free to the academic community on the DR. PRODIS (DRugome, PROteome, and DISeasome) webserver at http://cssb.biology.gatech.edu/dr.prodis/. DR. PRODIS provides protein targets of drugs, drugs for a given protein target, associated diseases and side effects of drugs, as well as an interface for the virtual target screening of new compounds.

Recent studies on the intrinsic characteristics of protein ligand binding pockets find that there is a limited number (~1,000) in nature^1^,^2^, whereas the number of proteins in a typical proteome (e.g., in human ~20,000) is far larger. The implication is that a given protein target binds many ligands, and conversely, a ligand binds many proteins, all with similar pockets^3^,^4^. Thus, the intrinsic promiscuity of a drug is partly responsible for its unintended side effects^5^, but this also suggests that FDA approved drugs could be utilized for large scale repurposing. That is, a drug could bind to another protein associated with a disease other than its intended target. Indeed, repurposing of FDA approved drugs for new indications is an efficient and accelerated means of drug discovery with applications to personalized medicine^5^,^6^.

Advances in whole genome sequencing^7^ make the identification of drug-target interactions more attractive and useful^5^. In practice, most DrugBank drugs (including FDA approved & experimental) have only a single or very few identified protein targets^8^. Thus, for many drugs, their possible targets are unknown. To fully explore drug and protein target promiscuity, given an arbitrarily identified disease-causing protein target, one should find all its binding drugs and side effects. To achieve this, screening of all plausible drugs against the human proteome and predicting side effects of an arbitrary drug are necessary. In contrast to genome sequencing technology that allows for the rapid identification of disease associated targets, brute-force experimental screening of all FDA approved or experimental drugs against a large number of identified disease associated protein targets is currently infeasible^5^,^9^. This work attempts to achieve this goal through a computational approach.

In parallel to experimental drug repurposing approaches^5^, many bioinformatics and computational approaches for drug-disease or drug-target relation discovery have been published^3^,^10^,^11^,^12^,^13^,^14^,^15^,^16^,^17^,^18^,^19^,^20^,^21^,^22^,^23^,^24^,^25^. Most exploit the similarity between drugs^17^, proteins^18^, side effects^19^, interaction network^12^ and diseases^10^. For example, for a given protein target, the chemical similarity of a drug to its known binding ligands was employed to predict possible association to a given protein target^17^,^25^. These methods require *prior* knowledge of a target protein’s or drug’s binding partners, side effects, interaction network, etc. There are also databases that collect experimental drug-protein interactions from the literature^3^,^26^. While a good idea in principle, in practice, the coverage of ligand and protein space of the above bioinformatics and computational methods are quite limited. Therefore, such methods are not yet applicable to the majority of the human proteome.

To address the limitation imposed by the requirement of *prior* knowledge of small molecule ligand-protein interactions, recent developments infer interactions from neighbors (evolutionarily related proteins)^27^,^28^ where such interactions are known. However, they have not yet been tested on a large scale, e.g. on DrugBank drugs^8^ when there are no known interactions for a given drug or a target of interest. Moreover, their 5 or 10 fold or leave-one-out cross validation (LOOCV) tests^27^,^28^ are dominated by drugs or targets with known interactions in their training library. Alternatively, as proposed earlier^20^, ref. 9 reports an ambitious initiative that applies a traditional structure-based docking approach to rank drug-target interactions by utilizing Google cloud computing. It extends target coverage to those proteins without *prior* knowledge of binding ligands. However, this approach is still limited by the requirement of having high-resolution target protein structures (available for at most only 1/3 of the human proteome^9^), and a lack of accurate scoring functions to rank docked ligands^29^,^30^.

Our recently developed and experimentally validated FINDSITE^comb^ ligand homology modeling approach^31^,^32^ has the following advantages over other state-of-the-art methods for predicting drug-protein interactions: (1) it does not require known interactions for a drug or protein target; (2) it does not require experimental or high resolution protein structures; (3) it is more efficient than traditional docking methods, and most importantly, (4) it has better accuracy for ranking drug-target interactions than traditional docking methods^31^. In practice, since FINDSITE^comb^ eliminates the prerequisite of having known binders and high-resolution protein target structures, it can screen ~86% of the protein sequences of a typical proteome whose structures can be reliably modeled. Once a library of target protein structures of a proteome is built, (e.g. the human proteome has around 20,000 unique proteins), virtual screening of a drug across the proteome only takes a couple of hours on a single CPU node. Thus, it is possible to predict interactions for millions of compounds against a typical proteome on a medium size computing cluster in a very short time.

Since the majority of human proteome targets have no known binders, in this work, we first focus on predicting drug-target interactions when neither the drug nor protein target has known binders, termed new drug and new target, respectively. This is the biologically important regime where many state-of-the-art approaches, such as SEA^25^, BLM^21^,^22^ and network methods^24^, are inapplicable. Besides FINDSITE^comb^ only a few methods, e.g. the machine learning BLM-NII^28^, the network-based^27^ and docking based algorithms^9^,^20^, can be applied. Here, we first benchmark FINDSITE^comb^ on a large set constructed from DrugBank^8^, and compare its performance against a new machine learning method BLM-NII^28^. We show that FINDSITE^comb^ has much better performance than BLM-NII as assessed by their AUC (area under the ROC curve) and Enrichment Factor (EF). We then apply FINDSITE^comb^ to screen all DrugBank drugs against the human proteome to discover new protein targets that mostly have no previously known or predicted ligand interactions.

We next turn to an examination of drug side effects, an indispensable aspect of drug discovery. While there are computational studies that assign side effects to protein targets and predict drug side effects^25^,^33^,^34^,^35^, their precision has not been systematically benchmarked. In fact, they require that their protein targets bind at least five known drugs with experimentally determined side effects^33^. Thus, they cannot infer side effects for the majority of human targets lacking experimentally known drug-protein interactions. To address these issues, drug side effects are inferred from predicted targets whose associated side effects are extracted from known drug-side effect relations using an empirical rule. Then, a *killing index*, κ, that predicts the likelihood of serious side effects is introduced. We show that κ correlates with an approved drug’s probability of being withdrawn, illicit and investigational, classifications often due to serious side effects. Thus, these predictions offer the promise of discovering potential new targets for and side effects of existing or new drugs.

## Results

**Figure 1:**
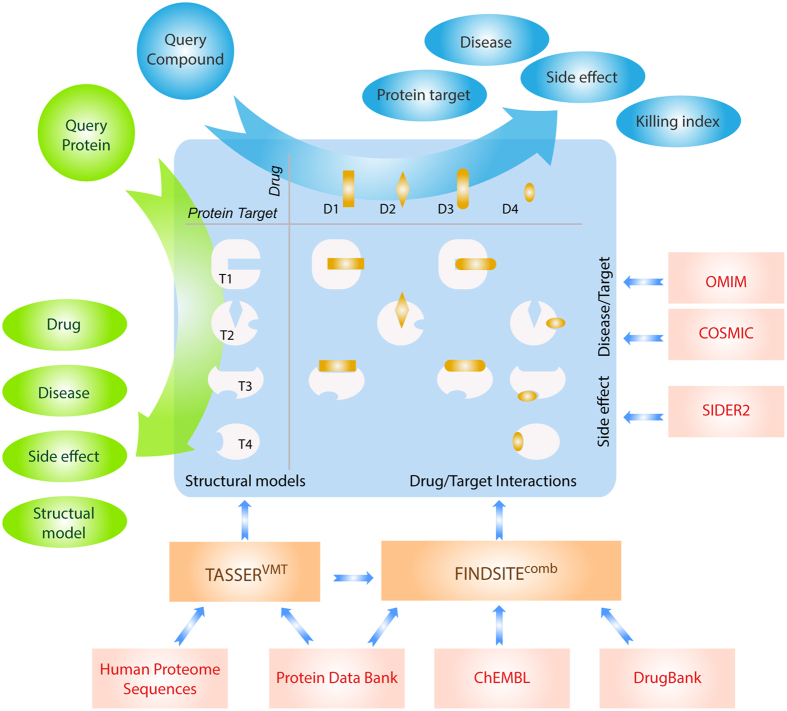
An illustration of the DR. PRODIS approach.

Our goal is to benchmark and present the DR. PRODIS knowledgebase shown in Fig. 1. The central idea is to infer the properties of a target drug or a target protein, such as their interaction partners, associated diseases and side effects for a drug from similar drugs or proteins (that may in fact be evolutionary very distant) found in databases. At the heart of the knowledgebase is the interaction data between proteins and small-molecule compounds. The data is predicted by FINDSITE^comb^. Thus, for the human proteome, we first evaluate the performance of the FINDSITE^comb^ for its ability to predict drug-protein interactions and compare its performance against one of the best extant methods, BLM-NII^28^. We then present results for the virtual target screening of DrugBank drugs against the human proteome and describe promising examples of drug repurposing to treat a variety of diseases. We next examine the ability to predict drug and protein target side effects across the human proteome. Finally, we combine predictions and known information about predicted drug-target interactions and drug and target side effects in the DR. PRODIS webserver and knowledgebase.

### Prediction of drug-protein interactions

#### Comparison of FINDSITE^comb^ with BLM-NII on the DrugBank set

We undertook a large-scale test of the ability to predict DrugBank drug-target interactions. To create a realistic scenario, we exclude interactions of targets and drugs from the library that have a sequence identity > a specified cutoff to the given target or that have a Tanimoto Coefficient^36^, TC > 0.99 to a known small molecule binder (this can exclude molecules that differ very little to the given drug). Since molecules having a TC > 0.85 likely have similar bioactivity^37^, a TC cutoff of 0.99 could possibly just include many molecules having similar bioactivity to the testing drug. However, as pointed in ref. 38 the TC = 0.85 cutoff myth is not generally true. Furthermore, in the tested DrugBank data, pairwise drug TC values within a given protein target range from 0.28 to 1.00, with an average of 0.61. Only about 14% of the molecules have a pairwise TC > 0.85. Thus, in practice a TC cutoff of 0.99 does not result in the majority of molecules having guaranteed similar bioactivity to the template drug that is used as a seed in virtual screening. On ranking the targets of a given drug, we first assess the results by the AUC as calculated per drug. We note that most drugs only have a few true targets in our test set (3,814 or 68% of the 5,639 DrugBank drugs have only one target). Thus, we use the Enrichment Factor (EF) as defined similar to that used in virtual ligand screening^31^ to assess performance:


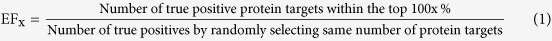


*EF*_*x*_ is the enrichment factor within the top **x** fraction (or 100**x**%) of screened targets relative to random selection. A true positive is an experimentally known binding target protein. That is, here, a protein target found among the drug-target relationships annotated in DrugBank for the given drug based on published experimental data. For **x** = 0.01, EF_0.01_ ranges from 0 to 100 (100 means that all true positives are within the top 1% of the screened targets). A value of EF_x_ > 1 means the method is better than random.

Table 1 compares FINDSITE^comb^ with SVM BLM-NII on the 5,639 DrugBank drugs for predicting protein targets with two different sequence identity cutoffs, 95% and 30%. The 30% cutoff assesses the ability to predict protein targets when no closely homologous templates are used for interaction inference. At a 95% sequence identity cutoff, FINDSITE^comb^ with an average AUC = 0.824 is significantly better than SVM BLM-NII, whose average AUC = 0.628. The number of drugs whose AUC is better than random (AUC = 0.5) by FINDSITE^comb^ is 4,948 (88%) compared to 3,798 (67%) by SVM BLM-NII. FINDSITE^comb^ has an EF_0.01_ enrichment factor of 38.05 vs. EF_0.01_ = 4.74 by SVM BLM-NII. Within the top 1% of the 3,576 screened targets, FINDSITE^comb^ has 2,583 (46%) drugs having at least one true target compared to 354 (6.3%) from SVM BLM-NII. Table 1 also includes the average rank of true targets: 627 for FINDSITE^comb^ vs. 1333 for SVM BLM-NII, respectively. While the performance of both methods diminishes when a 30% sequence identity cutoff is imposed, the performance of FINDSITE^comb^ is still significantly better. FINDSITE^comb^ has an EF_0.01_ = 14.04, with 1117 drugs having results better than random versus SVM BLM-NII which has an EF_0.01_ = 4.36, with 332 drugs better having an EF_0.01_ better than random.

One of many examples of a successful prediction by FINDSITE^comb^ is for DB00231 (*Temazepam*) that has 20 known targets. When a 95% sequence cutoff is applied, all 20 known protein targets are predicted within the top 1% (top 36 of 3,576) targets. When a 30% sequence cutoff is used, 17 of the known targets are within the top 1%.

#### Dependence of FINDSITE^comb^ ‘s performance on the mTC cutoff value

Here, we examine the dependence of precision and recall on the mTC cutoff value with precision and recall defined as


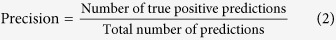



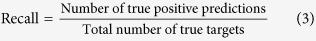


**Figure 2:**
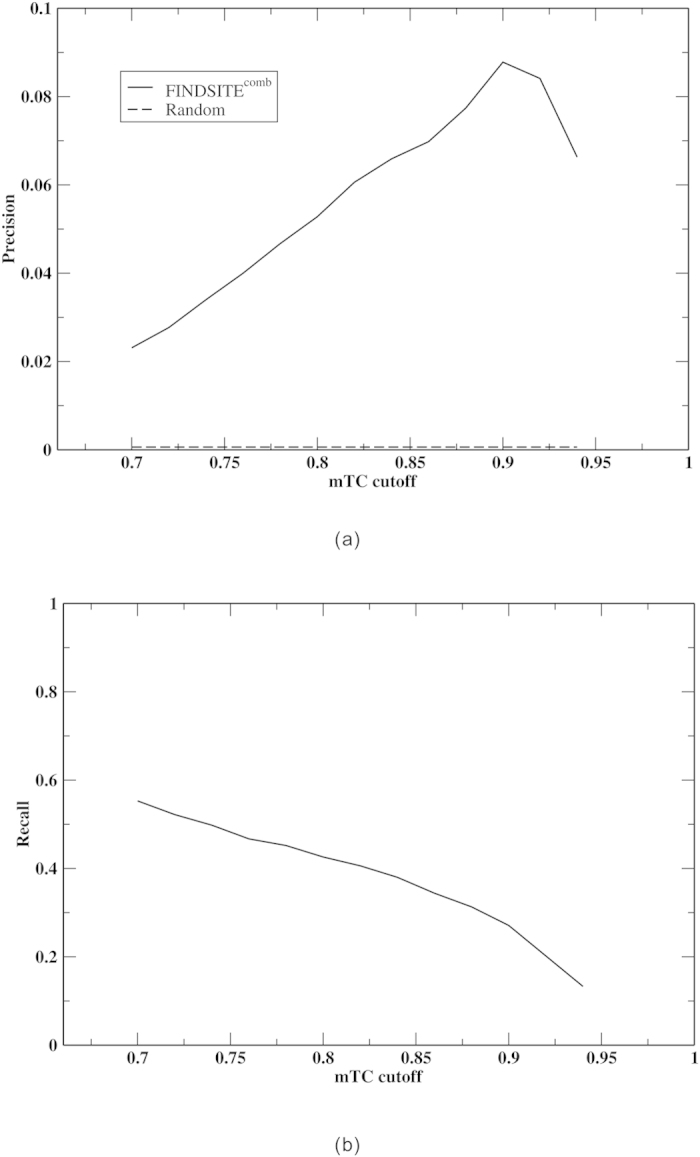
Average per drug: (**a**) precision vs. mTC cutoff; (**b**) recall vs. mTC cutoff for the DrugBank set in benchmarking mode.

Figure 2a,b show the average per drug relationship between precision and recall and the mTC cutoff for a target sequence cutoff of 95%. When mTC cutoff exceeds a given (high) threshold, some drugs will have no predicted protein target; thus, their contributions to the average abruptly drop to zero and the average precision starts to drop as well.

**Figure 3:**
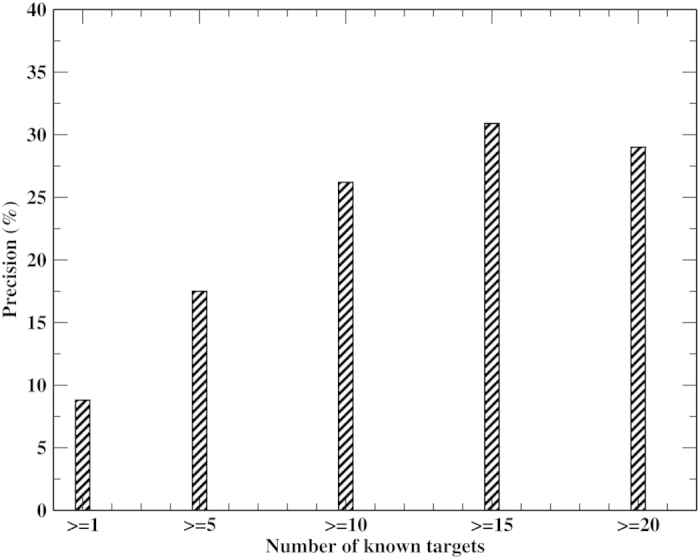
Dependence of “observed” protein target prediction precision on the number of known targets at mTC cutoff = 0.90 and 95% sequence cutoff.

Since we lack a complete list of all true drug targets, the precision shown in Fig. 2a is actually the “observed precision” rather than the true precision of the prediction. As shown in Supplementary Information, the more known targets a drug has, the more likely it is that the true and “observed” precision are close to each other. Therefore, to estimate the true precision, we should examine drugs having more known targets. Figure 3 shows the dependence of the “observed precision” on the number of known targets for an mTC = 0.90 (corresponding to a *P-value* = 5.66 × 10^−3^, see Supplementary Information) and 95% sequence identity cutoffs. When the number of known targets ≥10, the “observed precision” approaches ~30%. This is about three times the observed precision in Fig. 2a. Thus, ~30% should be close to the true precision. It is also consistent with the average 30.6% precision found in ligand virtual screening on the 102 protein DUD-E benchmark set^39^ with a mTC cutoff of 0.9. 30% precision is significantly better than that by random selection (precision ~0.0632%). At a 0.90 mTC cutoff, from Fig. 2b, the recall is ~27%. At a moderate cutoff of 0.70, recall is 55%, but the observed precision falls to 2.3%. In Supplementary Information, a test on 51 molecules against the yeast proteome shows similar results.

#### Virtual target screening of DrugBank drugs against the human proteome

The benchmark on the DrugBank set (as well as a “gold standard” set shown in Supplementary Information) shows that FINDSITE^comb^ is more accurate than BLM-NII for virtual target screening. We next apply FINDSITE^comb^ to screen DrugBank drugs across the entire human proteome to facilitate the discovery of new uses of existing drugs. We built a target structure library consisting of 97% (32,579) of all human proteins from the NCBI database (see Methods). We excluded exceedingly long protein sequences with more than 2,500 residues or sequences >1000 residues that cannot be parsed into smaller segments. Of these, 27,896 (86%) proteins have at least one segment modeled with a predicted TM-score ≥ 0.4^40^,^41^, a threshold when the predicted structure is significantly similar to the corresponding native structure (*P*-value of 3.4 × 10^−5^)^42^. In this regime, virtual screening is likely to be successful^31^.

**Figure 4:**
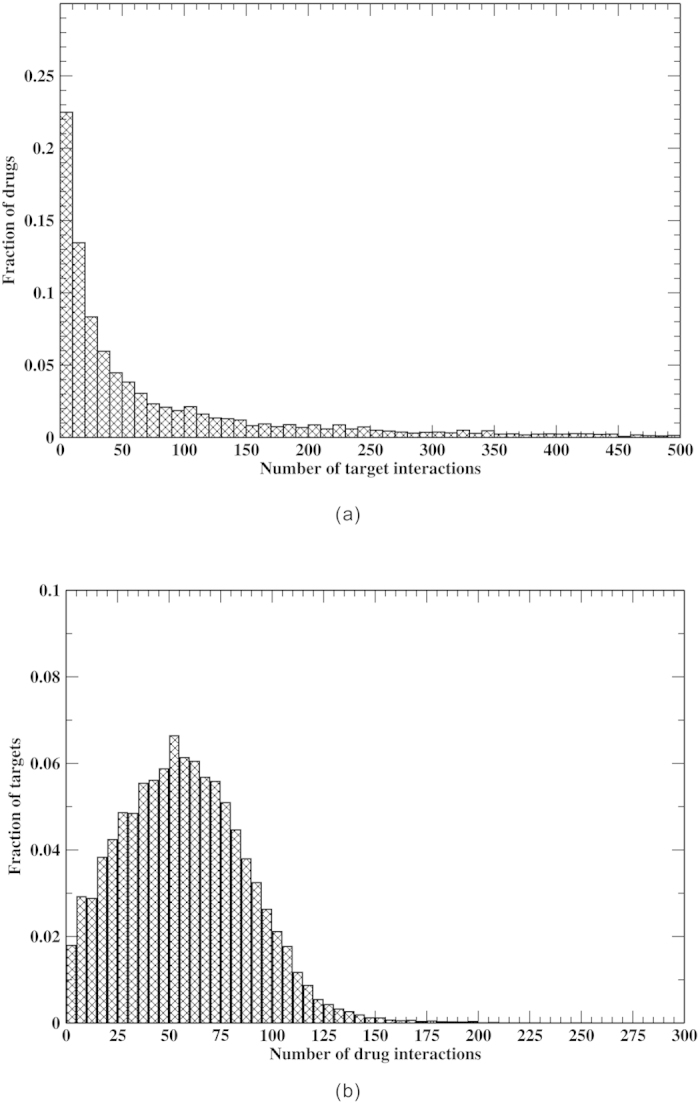
For the human proteome: (**a**) predicted drug distribution vs. the number of target interactions; (**b**) predicted target distribution vs. number of drug interactions for DrugBank drugs.

Next, all 5,639 DrugBank drugs were screened against this library in prediction mode (all interactions in the binding libraries are allowed). With an mTC cutoff of 0.90, the normalized distribution of drugs vs. the number of predicted interacting protein targets in the human proteome is shown in Fig. 4a. The average (median) number of predicted protein targets is 329 (38) per drug. Thus, most drugs likely have multiple targets. Similarly, Fig. 4b shows the distribution of protein targets vs. the number of interacting drugs. The average (median) number of drug interactions per protein is 57 (55). Thus, most proteins likely interact with many drugs. Finally, to avoid the effect of overrepresented targets in the target library, we clustered the protein targets into 13,404 clusters at a 30% sequence identity threshold. The distributions of drug-cluster interactions shown in Figure S1 are similar to Fig. 4. On average, a drug interacts with 141 protein clusters, and a protein target cluster interacts with 57 drugs.

#### Repurposing of drugs to treat new diseases

The above results once again demonstrate the likely promiscuity of drugs and protein targets found in our earlier work^1^. The promiscuity of a drug is particularly useful for repurposing it to treat new diseases. By the term “new disease”, we mean that the treatment of the particular disease is not the original intended use of the drug. Our predictions of new targets could potentially reveal new drug uses as well as side effects. On average, we predict 32.7 (140) genetic disease (somatic cancer) related targets for each drug. Of these only 7.4 (14.4) targets are identical to DrugBank targets (with ≥ 95% sequence identity). Around 80% (90%) of the predicted genetic disease (somatic cancer) related targets have not been considered as primary targets of existing drugs. Thus, our predictions could be useful for personalized medicine in the post genomic era when a disease (somatic cancer) is likely caused by mutations of a specific protein target, which does not have known drugs that restore the mutated protein’s molecular function to wild type.

Here, we present some successful examples of drugs predicted to target proteins involved in common and rare diseases. An antibiotic DB00997 (*Doxorubicin*) is commonly used to produce regression in disseminated neoplastic conditions like acute lymphoblastic leukemia and acute myeloblastic leukemia^8^. Our predictions show that it likely targets *hepatoma-derived growth factor* that causes *hepatocellular carcinoma*. Delcath Systems, Inc. has sponsored this as an orphan treatment of *hepatocellular carcinoma* (see http://www.fda.gov/ForIndustry/DevelopingProductsforRareDiseasesConditions/HowtoapplyforOrphanProductDesignation/ucm216147.htm). Drug DB01229 (*Paclitaxel*) is commonly used in the treatment of Kaposi’s sarcoma and cancer of the lung, ovarian, and breast^8^. Our predictions show that it binds to *BCL2-antagonist/killer 1* which causes *colorectal cancer, prostate cancer, pancreatic cancer*, and *tubulin, gamma 1* that causes *cortical dysplasia, complex, with other brain malformations*. According to the FDA web site, MediGene AG has sponsored it for treatment of pancreatic cancer and Protherics, Inc. for brain cancer.

While FINDSITE^comb^ is one of the best methods for predicting drug-protein target interactions, still the majority of its predictions (~70%) are likely false positives. Thus, it should be viewed as a “look here, not there” means of prioritizing which experiments should be done.

### Prediction of drug and target side effects across the human proteome

#### Benchmarking results

Using predicted human targets of a given drug and the inferred target-side effect relations, we predict the side effects of the given drug as the union of side effects from all of its protein targets. We first perform a consistency test by examining how well our inferred target-side effect relations can reproduce known input side effects: i.e. all known drug-side effect relations are utilized to build target/side effect relations, which is used to predict the input drug’s side effects. We then test the predictive power of our method in a jackknife test: All, but one, of a drug’s side effects are utilized to build target/side effect relations, we then predict the side effects of the left out drug.

To examine the usefulness of the FINDSITE^comb^ predicted drug-target relations in the inference of protein target side effects, the above tests are also carried out using only experimental drug-protein target binding information as provided by STITCH3^26^. The resulting average precision and recall for the 996 SIDER2 set^43^ are compiled in Table 2. In the first row of Table 2, we only use known (*viz*. experimental) drug-protein interactions, whereas in the second row we only use FINDSITE^comb^ predicted drug-protein target interactions with no cutoffs applied. The consistency test gives zero false positives due to the deterministic nature of the inference rule. Around 30% of side effects can be recalled using either predicted or experimental drug-target relations. The similarity of results lends additional confidence to our predictions of such drug-target interactions. Missed side effects reflect the incompleteness of target collection/prediction of the drug.

In the jackknife test, for either experimental known or predicted drug protein interactions, with respect to the consistency test, both precision and recall decrease. Comparing the use of predicted drug-target interactions with experimental drug-target interactions, there is only a slight drop in precision in the jackknife test from 58.4% to 56.5%, whereas recall *increases* from 16.6% to 23.6%! Using predicted drug-target relations also *increases* the number of drugs having predictions from 671 to 740. Again, the increase is likely due to fact that there are more correct drug-target relations, on average, present in the predicted than experimental relationships. Thus, using the FINDSITE^comb^ predicted drug-target relations gives a favorable contribution to drug side effect prediction. Although our drug side effect predictions give a binary classification and do not rank side effects, we note that at the same ~24% recall rate, the machine learning approach in ref. 35 has a precision of ~30% in its fivefold cross validation; much less than our precision of 56.5%.

In Table 2, we also compare results for reproducing input drug-side effects using experimentally determined drug-target relations and the statistical method described in ref. 33. With a conservative *P-value* cutoff of 1 × 10^−3^, the precision is only 19.1% with a recall of 58.3% due to the large number of false positive predictions by the statistical method. Decreasing the *P-value* cutoff does not significantly improve the precision, but recall decreases rapidly. Our method for reproducing the input has five times the precision, yet only reduces the recall by half.

#### Drug and target side effect predictions for the human proteome

**Figure 5:**
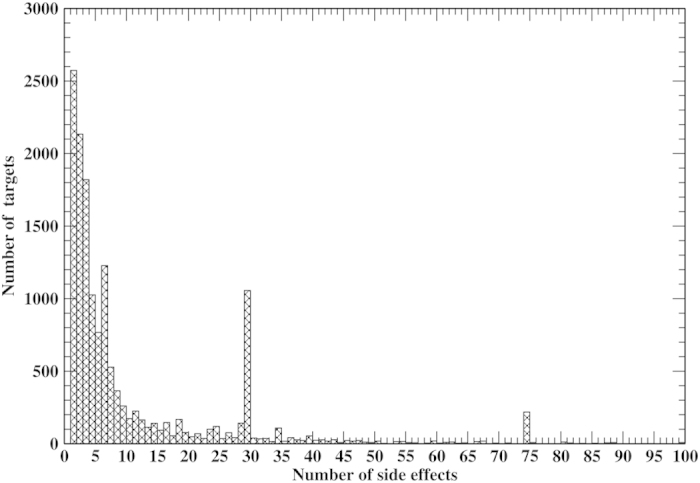
Number of protein targets vs. the number of side effects.

Using predicted drug-target relations and all experimental 996 drug side effects from SIDER2^43^, we inferred side effects for 14,934 human protein targets. The distribution of the number of side effects of a protein target is shown in Fig. 5. This distribution obeys a power law, consistent with the result from a statistical method^33^. 2,573 protein targets have one side effect. The majority of protein targets have ≤ 4 side effects. The average (median) number of side effects per protein target is 11.6 (3.5). Six targets have the maximum number of 339 side effects. Consistent with the recall rate of 23.6%, 877 of 4,192 distinct side effects appear in at least one target. The most frequent side effects are *nausea*, *vomiting*, *diarrhea*, appearing in 10,755, 9,255 and 7,515 targets, respectively.

Drug side effects are predicted for all DrugBank drugs using the above FINDSITE^comb^ predicted drug-target relations and the above inferred target-side effect relations. 4,975 drugs have side effect predictions, with the number of side effects ranging from 1 to 849. On average, 85 side effects are predicted for a given drug. An example of side effect prediction is presented for DB00136 (*Calcitriol*) that is used to treat vitamin D deficiency. Our method predicts 33 side effects, such as *anorexia, nausea, vomiting, polyuria, polydipsia, weakness and pruritus* that are consistent with DrugBank annotations. Another example is the DB00563 (*Methotrexate*), which is used for treating *gestational choriocarcinoma, chorioadenoma destruens and hydatidiform mole* (a rare mass or growth that forms inside the womb at the beginning of a pregnancy). We recover 114 (56%) of the 203 known side effects with 100% precision that are reported in the SIDER2 database. These include *sudden death* & *death*. Both side effects come from the drug’s interaction with protein target *transmembrane protein 222* encoded by the *TMEM222* gene. The function of this gene is, unfortunately, unknown.

#### Drug killing index

1,165 protein targets are inferred to have serious side effects such as *death, stroke, cancer*, and *heart failure*. Drugs that bind to these proteins will likely have serious side effects. Depending on whether the drug is an agonist or antagonist to the target, the serious side effect may or may not occur. 2,456 of 5,639 or 44% of molecules from DrugBank are predicted to have a *killing index* κ > 0, whereas only 192 of the 1,187 (or 16%) FDA approved drugs (non-nutraceuticals) have a κ > 0. Thus, κ can discriminate between an arbitrary small molecule and an FDA approved one. Eliminating drugs having κ > 0 still gives an 84% recall rate for FDA approved drugs. κ is highly correlated with promiscuity (the number of protein targets a drug binds to) with a Pearson’s Correlation Coefficient (CC) = 0.89.

**Figure 6:**
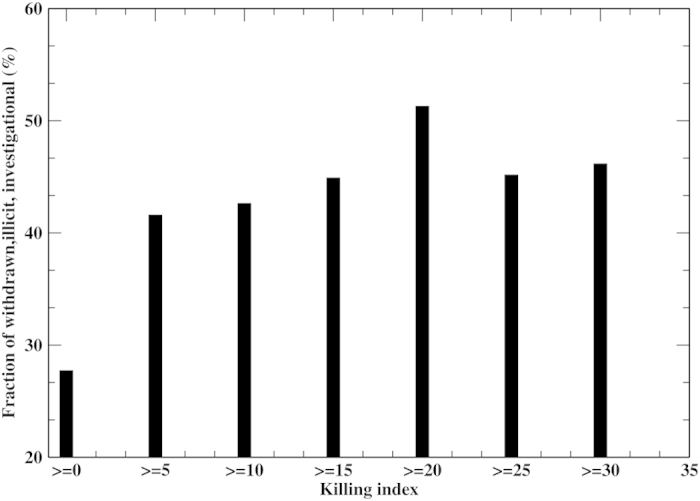
Dependence of the cumulative fraction of drugs being withdrawn, illicit & investigational on killing index.

To further show that the predicted *killing index* is meaningful, we present the relationship between the fraction of FDA approved drugs being withdrawn, illicit or investigational versus *killing index* in Fig. 6. The fraction of problematic drugs increases from 28% for all FDA approved drugs to 51% for approved drugs having a *killing index* ≥20. Thus, the *killing index* is correlated with the probability of an approved drug being withdrawn, illicit or investigational (CC = 0.75), that is usually related to serious side effects.

### DR. PRODIS drug, protein, disease and side effect webserver

A web service for FINDSITE^comb^ based virtual target screening has been implemented using the above human target protein library at http://cssb.biology.gatech.edu/dr.prodis/. To facilitate navigation of the search results, all interacting targets and drugs are URL cross-linked. The two major functionalities are: (a) an interface for searching the pre-computed DRugome, PROteome, and DISeasome (DR. PRODIS) knowledgebase constructed from the above virtual target screening of DrugBank drugs against Human proteome. For each protein target, DR. PRODIS provides information about disease-causing genetic mutations, somatic cancer driver mutations, inferred side effects, predicted bound DrugBank drugs, predicted protein structures and putative drug binding sites. For each DrugBank drug, the knowledgebase provides side effects, *killing index* and human protein targets. (b) virtual target screening for new compounds against the human proteome. A given drug’s 2D or 3D structure is required as input and the protein target library can be selected from *Human*, as well as three other proteomes, *p. Falciparum*, *m. Tuberculosis and yeast*, whose analyses are beyond the scope of this paper. Predictions are available for manual review on our web server or for download. If the human target library is selected, for each small molecule, its predicted side effects, *killing index*, and a URL link for each target to the DR. PRODIS database will also be provided. This service will be useful for discovering protein targets and possible side effects of potentially interesting molecules or for designed new drug molecules.

## Discussion

In this paper, we developed a comprehensive approach to predicting drug-protein interactions that allows us, with an acceptable precision and recall of ~30%, to predict for a given drug in DrugBank or a novel small molecule ligand, its possible side effects, *killing index*, and protein targets in the human proteome. Conversely, for a given protein in the human proteome, in the majority of cases, we provide its predicted structure and binding sites, predicted FDA approved and experimental drugs that might bind to the protein, possible side effects, as well as diseases associated with non-synonymous amino acid mutations. On average, we predict that a given drug binds 329 targets, and each protein binds about 57 drugs. Consistent with previous fundamental work on the number of distinct ligand binding sites^1^, we again find that promiscuous drug protein interactions are quite likely. Even if our predictions are off by a factor of 10, such promiscuity has to be accounted for in the process of drug discovery; but concomitantly, it can be used for the large scale repurposing of FDA approved drugs.

We next developed a simple approach to side effect prediction. Comprehensive benchmarks suggest that we can predict drug side effects with a precision of about 57% and a recall of about 24%. We also show that a drug’s promiscuity is highly correlated with the derived *killing index*, which in turn is correlated with a drug being FDA approved or being withdrawn if it is approved. The unification of the DRugome, PROteome, and DISeasome information is available to the academic community at the DR. PRODIS database and webserver.

Comparison with the state-of-the-art sequence-based machine learning approach BLM-NII^28^ for drug-protein target interaction prediction shows that FINDSITE^comb^ has consistently better performance. Most existing methods such as the SEA^25^, BLM^21^, network^24^ and many others^3^,^5^,^10^,^11^,^16^,^23^,^44^ cannot provide such comprehensive predictions due to their requirement of known interactions.

Competing traditional structure-based docking methods can provide protein target coverage for only 1/3 of human proteome and require very large scale cloud computing resources^9^. In contrast, FINDSITE^comb^ covers 86% of the human protein, is far more computationally efficient and is applicable to predicted as well as experimental structures. Most importantly, FINDSITE^comb^^31^ performs significantly better than structure-based docking methods for ligand virtual screening on the relatively small DUD benchmark set^45^. We would expect that FINDSITE^comb^ also performs better than docking methods for protein target virtual screening^9^.

With the above advantages in mind, there are also disadvantages of current knowledge based approach that employs the ideas of homology modeling applied to ligand identification: (a) it cannot predict absolute binding affinity. Rather, the predicted drug-protein interactions are meaningful for relative ranking. (b) There are no ligand bound poses in the DrugBank & ChEMBL binding libraries; rather they merely provide information as to which ligands bind which template. Thus, for this component of FINDSITE^comb^, we can infer which ligands likely bind to the target protein but not necessarily the binding pose. In contrast, when the binding template is from the PDB, binding poses are predicted as in FINDSITE^46^,^47^. We further describe the procedure to predict such poses along with some examples in Supplementary Information. We are currently working on addressing both limitations.

In summary, we have developed a comprehensive approach to drug-protein target-disease-side effect prediction that while not perfect, has sufficient predictive value to guide experimental studies, and clinical repurposing of FDA approved drugs. It should be noted that the predicted drug-protein interactions, side effects & killing index provided by the current approach, as well as any other bioinformatics tool should serve as guides as to which experiments should be done rather than absolute rules.

## Materials and methods

### Preparation of target and template libraries for FINDSITE^comb^

#### Modeling of the structures in the human proteome

To apply FINDSITE^comb^ for predicting unknown targets of a drug by virtual target screening, we built a target library consisting of structural models of the human proteome (from ftp://ftp.ncbi.nih.gov/genomes/H_sapiens/protein/, early 2012). To model long multi-domain proteins, we divide their sequences into smaller segments (each segment itself could contain multiple domains) using the automated sequence parsing procedure shown in Figure S2. After parsing each target sequence, the structure of each segment is independently modeled using TASSER^VMT^-lite^41^. The top ranked model, given by SPICKER clustering^48^ on the low energy trajectories from the TASSER simulation^49^, is the predicted structure for each segment.

In practice, we built structure models for 32,579 human proteome protein targets. Of these, 27,896 or 85.6% have at least one segment with a predicted TM-score^40^ to native ≥ 0.4. The TM-score is a structural similarity measure with values between 0 and 1^40^. Two proteins are structurally related if they share a TM-score ≥ 0.40 (*P-value* of 3.4 × 10^−5^)^42^. Earlier, large scale, ligand virtual screening benchmarking shows that for a target with a model TM-score ≥ 0.4 to native, FINDSITE^comb^ gives a better enrichment factor than random selection^31^.

### Methods for predicting drug-protein target interactions

#### FINDSITE^comb^

Our previously developed and experimentally validated FINDSITE^comb^ ligand homology approach is used to predict possible drug-target interactions^31^,^32^. For a given drug/compound with a 2D or 3D structure and a target protein sequence or experimental structure, three scores are computed independently by: (1) FINDSITE^filt^^50^ that infers binding sites and ligands of a target from threading identified holo proteins in the PDB database that have bound ligands; (2) FINDSITE^X ^41 that improves performance for those targets with few or no threading identified holo PDB templates by predicting the structures of virtual holo templates and extracting their known ligands from the ChEMBL^51^ and the DrugBank^8^ drug-target databases.

For each component, comparison of the inferred representative ligand set of the target (since they are inferred from template, they are called “template ligand”) to the input drug/compound is carried out using the mTC score defined by:


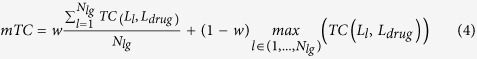


where *N*_lg_ is the number of template ligands from representative set; TC is the Tanimoto Coefficient^52^ of two 1,024-bit fingerprints^53^,^54^ from the template ligand and drug/compound, respectively. *L*_*l*_ and *L*_*lib*_ are the template ligand and drug/compound, respectively; *w* is a weight parameter. We set **w** = 0.1 to give more weight when the template ligands are true ligands of the target. The best score from each of the three component approaches is selected.

#### Structure-pocket and structure-structure comparison procedures

In FINDSITE^comb^, as mentioned above, template ligands are inferred from similarities of structure-pocket or structure-structure comparisons between target and template proteins. A pocket structure of a given PDB template consists of the C_α_ atoms of residues, any of whose heavy atoms lie within 4.5 Å of the ligand heavy atoms and additional C_α_ atoms that are found within 8 Å of the ligand heavy atoms. A heuristic alignment method^31^ is employed for structure-pocket comparison: (1) exhaustive comparison of sequence order dependent triplets of C_α_ atoms of the target structure and the template pocket; (2) if the corresponding triplet distances are within 1 Å of each other, the corresponding 3 residues are used as a seed alignment to do an optimal superposition by minimizing the root mean squared deviation (RMSD); (3) after this structural superposition, those residues within 1 Å of each other in the target and the template become the new seed alignment; (4) the alignment procedure is iterated until the set of aligned residues is unchanged. The alignment is ranked by a score depending on both structure and sequence similarities of the aligned residues. Structure-structure comparison is done using fr-TMalign^55^ ranked by summation of the BLOSUM62 substitution matrix^56^ scores over the aligned residues.

Here, we need to compare a target of multiple segment structures (domains) to a library PDB pocket or a library template of multiple segments. The best scoring pocket for each target-template segment pair is chosen for ranking. The problem is that a single target segment could in principle dominate pocket selection. A better procedure would include all target protein segments and their identified template ligands. This more general approach is currently being examined^57^. Another issue is that a pocket might only exist when the global fold of all target/template segments form^58^, e.g. it can be created when two protein domains pack against each other. The consequence of these approximations is that our predicted number of protein targets per drug is likely a lower bound.

#### BLM-NII Implementation

To compare the performance of FINDSITE^comb^ with BLM-NII^28^ to predict drug-target interactions, when both drug and target have no known interactions, we implemented our own, improved version of BLM^22^ (bipartite local model). Because BLM cannot predict interactions between new drugs and new targets, BLM-NII uses neighbor information to extend its applicability. We used the same drug and target similarity matrices downloaded along with the gold standard set (see Supplementary Information) and employed SVM^light^ (http://svmlight.joachims.org/) Support Vector Machine (SVM) regression^59^ to train the local models for each drug-target interaction. SVM is used in the original BLM^22^ method, whereas a regularized least squares (RLS) is used in BLM-NII^28^. Ref. 28 includes a network-based similarity matrix whose contribution can be controlled. In our implementation, for simplicity and efficiency, we do not use the network-based similarity matrix.

### Benchmark sets for the assessment of drug-protein interactions

#### DrugBank benchmark set

To test methods in a realistic target virtual screening scenario, i.e., finding the true targets of given drug from a library of targets, we use a large set constructed from all DrugBank drug-target relations that contain interactions between 5,639 (1,250 FDA approved + 4,389 experimental) drugs and 3,576 targets for which we can model, and 12,744 known drug-target interactions. Only 256 drug-target pairs are singletons (both drug and target have only one known interaction). We artificially make all interactions singletons for benchmarking purposes; that is, we exclude from the binding libraries or training set all known interactions of the drug and protein target whose interaction is being predicted.

### Drug and target side effect predictions

#### Drug & target side effects

A particular drug side effect is assumed caused by binding to a particular protein target, with the totality of drug side effects being the sum of all their protein target side effects. That is, if protein target (T_1_) binds to drugs (D_1_, D_2_, …, D_k_)(k > 1), all sharing common side effects (S_1_, S_2_, …, S_n_), then these side effects are associated with protein T_1_. Once protein-side effect relations are inferred, they are used for predicting the side effects of a target drug. The predicted side effects of a drug are the union of side effects from all its binding targets.

Protein target side effects are inferred from drug-side effect relations provided by the SIDER2 database (http://sideeffects.embl.de/)^43^. SIDER2 has 99,423 drug-side effect pairs involving 996 drugs and 4,192 distinct side effects (~100 side effects/drug). Predicted drug-target relations are provided by FINDSITE^comb^. Experimental drug-target binding information obtained from the STITCH3 database (http://stitch.embl.de/)^26^ are used to benchmark the approach.

#### Killing index of a drug

To quantify the likelihood of drug having toxic side effect, we define the *killing index* of a drug as its number of targets with serious side effects. These side effects are: *death, sudden death, sudden cardiac death, cardiac death*, *cancer, hemorrhagic strokes, heart failure, and congestive heart failure*.

### Data Availability

All benchmark data sets, structural models of the human proteome, and the results of our method as applied to the benchmark sets and the human proteome are available at http://cssb.biology.gatech.edu/dr.prodis/.

## Additional Information

**How to cite this article**: Zhou, H. *et al*. Comprehensive prediction of drug-protein interactions and side effects for the human proteome. *Sci. Rep*. **5**, 11090; doi: 10.1038/srep11090 (2015).

## Supplementary Material

Supplementary Information

## Figures and Tables

**Table 1:**
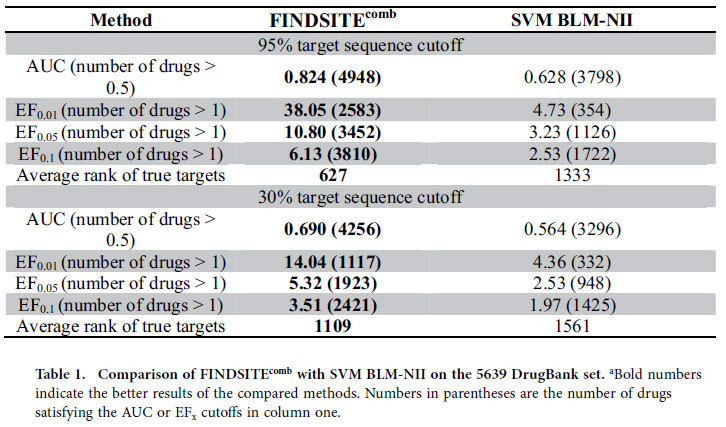
Comparison of FINDSITE^comb^ with SVM BLM-NII on the 5639 DrugBank set.

**Table 2:**
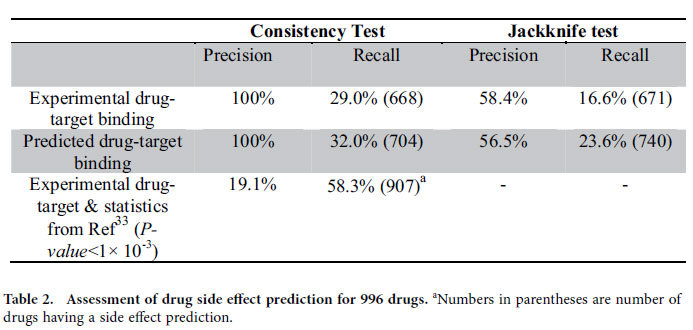
Assessment of drug side effect prediction for 996 drugs.
