# Supplementary material for: Comprehensive prediction of drug-protein interactions and side effects for the human proteome
Source: Sci Rep. 2015 Jun 9;5:11090. doi: 10.1038/srep11090 (PMC4603786; doi:10.1038/srep11090)
Supplement: Supplementary Information [file srep11090-s1.pdf]

SUPPLEMENTARY INFORMATION

for

Comprehensive prediction of drug-protein interactions and side effects for the human proteome

Hongyi Zhou, Mu Gao and Jeffrey Skolnick

Gold Standard set for the assessment of drug-protein interactions

The "gold standard" drug-protein target benchmark set collected by Yamanishi *et. al.* in Ref [1] from KEGG BRITE[2], BRENDA[3], and DrugBank[4] (including the drug-target similarity matrices) was downloaded from http://www.lmmd.org/database/dti/. In practice, it consists of four subsets: Enzyme, Ion-channel, GPCR and Nuclear-receptor, whose statistics are given in Table S1.

Comparison of FINDSITE^{comb} with BLM-NII on the gold standard set

We first examine if our implementation of BLM-NII is correct so that we can compare to this approach in variety of contexts, using the AUC (area under the ROC curve) and AUPR (area under the precision-recall curve) criteria. Table S2 shows that our implementation has very similar performance to the original RLS BLM-NII[5] (α=1, g=max) in the leave-one-out cross validation (LOOCV) test on the gold standard set, where known drug-protein target interactions are allowed. Next, more realistic tests were undertaken. In Table S3, we compare the performance of SVM BLM-NII with FINDSITE^{comb} in a scenario where neither the drug nor target protein has known drug-protein interactions. Here, interactions of the given pair to other targets/drugs are excluded from the SVM BLM-NII local model training and the FINDSITE^{comb} binding libraries (i.e. PDB, ChEMBL & DrugBank). FINDSITE^{comb} gives consistently better results across all subsets as assessed by both AUC and AUPR criteria. In particular, for the Enzyme, Ion-Channel and GPCR subsets, the AUPR given by FINDSITE^{comb} is more than twice that of SVM BLM-NII. Even though the precision is somewhat low, the fact

that target screening is better or much better than random demonstrates the usefulness of these methods as a pre-filter for experimental target screening.

Test on yeast proteome

Yeast (*Saccharomyces cerevisiae* S288c) proteome sequences were downloaded from ftp://ftp.ncbi.nlm.nih.gov/genomes/. A target library of 5,770 predicted protein structures was built. We then screen 3,356 molecules from the HIPHOP database that were identified to inhibit wild-type yeast growth[6] against this library. Using an mTC cutoff of 0.9, we predict targets for 528 molecules with average number of 39.6 targets per molecule and an average number of 6 molecules per target. We next evaluated the performance of FINDSITE^{comb} on a subset of 51 molecules that have experimentally established target information[6]. The "observed precision" and recall are 9.2% and 46.1%, respectively, results very similar to that in large-scale benchmarking results for human proteome.

Relationship of "observed" to true precision

When only N_{obs} of N_{true} proteins targets are known and the predicted number of correct protein targets is N_{pred}^{corr} of a total of N_{pred} predictions, then the number of "observed" true positive predictions will be $N_{obs}^{corr} = N_{pred}^{corr} \times (N_{obs} / N_{true})$. The "observed precision" is given by $precision = N_{pred}^{corr} \times (N_{obs} / N_{true}) / N_{pred}$ Thus, the true prediction precision $N_{pred}^{corr} / N_{pred}$ is the ratio of the "observed precision" $/(N_{obs} / N_{true})$, and only when N_{obs} is close to N_{true}, will the true precision equal the "observed precision"; this is

more likely to hold the greater is the number of known targets. Note that the "observed recall" rate does not depend on N_{obs} and is the same as true recall rate.

Determination of the *P-value* for a given mTC score.

Using all the possible 20,152,320 drug-target pairs from DrugBank (3,576 × 5,639-12,744, with 12,744 the number of known drug-protein target interactions) that are not known true positive interactions as random pairs to derive the random mTC score distribution, we find that the extreme value distribution (EVD)

$$y = f(x \mid \mu, \sigma) = \sigma^{-1} \exp\left(\frac{\mu - x}{\sigma}\right) \exp\left(-\exp\left(\frac{\mu - x}{\sigma}\right)\right) \qquad \text{(S1)}$$

fits well, with parameters $\mu = 0.54502,\ \sigma = 0.06863$. The *P-value* of mTC=$x_0$ is the probability of mTC>x_0 by chance and is calculated using

$$P - value(x_0) = \int_{x_0}^{1} f(x \mid \mu, \sigma)\,dx = 1 - \exp\left[-\exp\left(\frac{\mu - x_0}{\sigma}\right)\right] \qquad \text{(S2)}$$

An mTC value of 0.9 corresponds to a *P-value*=5.66×10^{-3}, whereas mTC=0.7 corresponds to a *P-value*=0.099.

Mapping of diseases to proteins

To identify protein targets associated with genetic diseases or cancers caused by mutations, each target is mapped to the genes that have disease-causing genetic mutations obtained from UniProt (http://www.uniprot.org/docs/humsavar), which in turn is derived from the OMIM database[7,8]. Cancer related somatic mutation data were downloaded from the COSMIC database (ftp://ftp.sanger.ac.uk/pub/CGP/cosmic/)[9], with mutations that occur in at least in two samples assumed to be driver mutations. We then mapped our protein library targets to proteins containing driver mutations.

Binding pose prediction

For drug-protein interactions whose structural template contains a protein-ligand complex, we can predict the drug-protein binding pose by superimposing the target protein structure to the pocket of the template protein structure and superimposing the putative drug structure to the template ligand structure, respectively. The ligand superimposition is conducted using the ligand alignment method LIGSIFT[10]. Since the protein target is modeled without the drug and is not a perfect prediction, clashes between the protein and drug are often inevitable. Nevertheless, important information can be inferred from this approximate binding pose: where the drug binds, what residues could be in contact with the drug, etc. Figure S3 shows two examples of predicted binding poses by FINDSITE^{comb}.

References

1 Yamanishi, Y., Araki, M., Gutteridge, A. & Honda, W. Prediction of drug-target interaction networks from the integration of chemical and genomic spaces. *Bioinformatics* **24**, i232--i240 (2008).

2 Kanehisa, M., Goto, S., Kawashima, S., Okuno, Y. & Hattori, M. The KEGG resource for deciphering the genome. *Nucl. Acid. Res.* **32**, D277-280 (2004).

3 Schomburg, I. *et al.* BRENDA, the enzyme database: updates and major new developments. *Nucl. Acid. Res.* **32**, D431-D433 (2004).

4 Wishart, D. *et al.* DrugBank: a comprehensive resource for in silico drug discovery and exploration. . *Nucl. Acid. Res.* **34**, D668-672 (2006).

5 Mei, J., Kwoh, C., Li, X. & Zheng, J. Drug-target interaction prediction by learning from local information and neighbors. *Bioinformatics* **29**, 238--245 (2013).

6 Lee, A. *et al.* Mapping the cellular response to small molecules using chemogenomic fitness signatures. *Science* **344**, 208--211 (2014).

7 Hamosh, A. *et al.* Online Mendelian Inheritance in Man (OMIM), a knowledgebase of human genes and genetic disorders. *Nucl. Acids Res.* **30**, 52--55 (2002).

8 Consortium, T. U. Activities at the Universal Protein Resource (UniProt). *Nucl. Acid. Res.* **42**, D191-D198 (2014).

9 Bamford, S. *et al.* The COSMIC (Catalogue of Somatic Mutations in Cancer) database and website. *British Journal of Cancer* **91**, 355--358 (2004).

10 Roy, A. & Skolnick, J. LIGSIFT: An open-source tool for ligand structural alignment and virtual screening. *Bioinformatics* **in press** (2015).

Table S1

Statistics of the drug-target interaction "gold standard" benchmark set.

Subset	Enzyme	Ion-channel	GPCR	Nuclear receptor
Drugs	445	210	223	54
Targets	664	204	95	26
Interactions	2926	1476	635	90
Drug has a single target	177	81	106	39
Target has a single drug	288	23	34	8
Both drug & target have a single interaction	14	1	8	3

Table S2

Comparison of SVM BLM-NII with RLS BLM-NII[5] on the gold standard set in the LOOCV test

**Subset**	**AUC**[a]		**AUPR**[a]	
	SVM BLM-NII	RLS BLM-NII(α=1, g=max)	SVM BLM-NII	RLS BLM-NII(α=1, g=max)
Enzyme	0.976	0.980	0.889	0.873
Ion-channel	0.979	0.978	0.762	0.846
GPCR	0.971	0.981	0.767	0.788
Nuclear-receptor	0.979	0.969	0.869	0.807

[a]AUC is the area under the ROC curve and AUPR is the area under the precision-recall curve.

Table S3
Comparison of $FINDSITE^{comb}$ with SVM BLM-NII on the gold standard set for new drug-protein interactions

**Subset**	**AUC**		**AUPR**		
	$FINDSITE^{comb}$	SVM BLM-NII	$FINDSITE^{comb}$	SVM BLM-NII	Random
Enzyme	**0.734**	0.681	**0.080**	0.038	0.010
Ion-channel	**0.690**	0.612	**0.138**	0.066	0.034
GPCR	**0.765**	0.729	**0.125**	0.059	0.030
Nuclear-receptor	**0.783**	0.723	**0.189**	0.136	0.064

[a] Bold numbers indicate the better of the two methods.

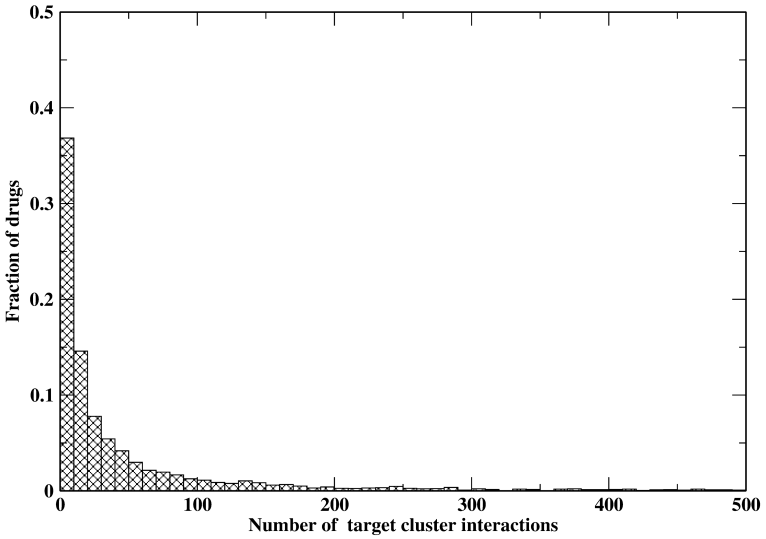

(a)

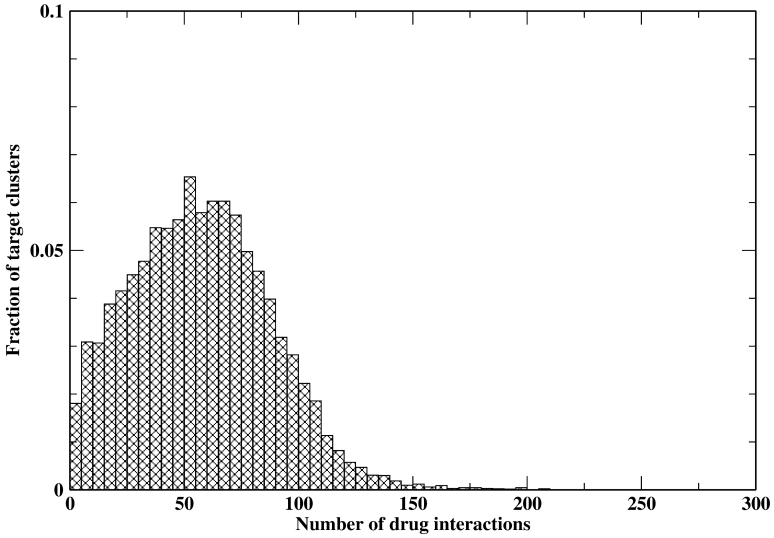

(b)

Figure S1: For the human proteome: (a) Predicted drug distribution vs. number of target cluster interactions; (b) Predicted target cluster distribution vs. number of drug interactions for DrugBank drugs screened against human proteome.

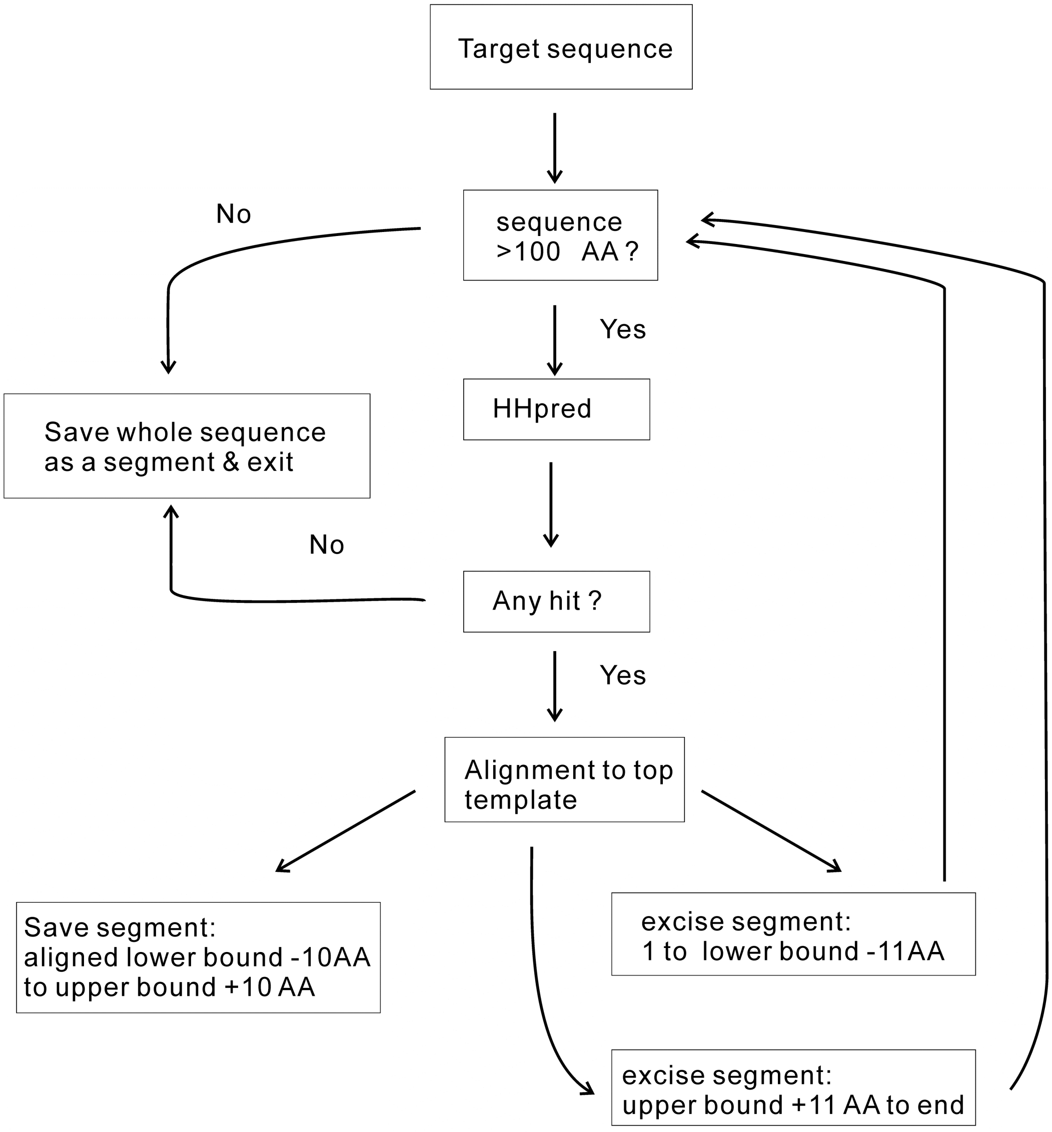

Figure S2: Target sequence parsing procedure. Upper (lower) bound is the aligned target position of the template N(C)-terminal.

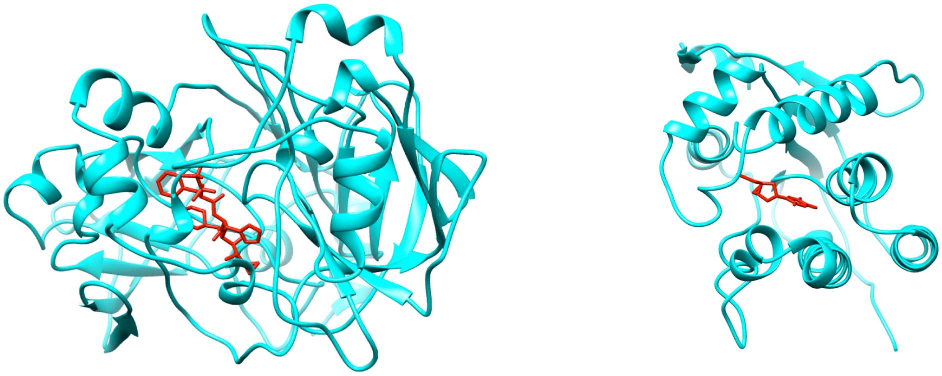

(a) (b)

Figure S3: Examples of predicted binding poses by FINDSITE^{comb} : (a) pepsin A preproprotein binding to drug DB00212 (*Remikiren*) based on template 3D91_B; (b) thymidine kinase 2, mitochondrial precursor binding to drug DB00495 (*Zidovudine*) based on template 2JJ8_D.
